# Risk of Hospitalization for Adverse Drug Events in Women and Men: A Post Hoc Analysis of an Active Pharmacovigilance Study in Italian Emergency Departments

**DOI:** 10.3390/ph14070678

**Published:** 2021-07-15

**Authors:** Giada Crescioli, Ennio Boscia, Alessandra Bettiol, Silvia Pagani, Giulia Spada, Giuditta Violetta Vighi, Roberto Bonaiuti, Mauro Venegoni, Giuseppe Danilo Vighi, Alfredo Vannacci, Niccolò Lombardi, on behalf of the MEREAFaPS Study Group

**Affiliations:** 1 Department of Neurosciences, Psychology, Drug Research and Child Health, Section of Pharmacology and Toxicology, University of Florence, 50139 Florence, Italy; giada.crescioli@unifi.it (G.C.); ennio.boscia@stud.unifi.it (E.B.); roberto.bonaiuti@unifi.it (R.B.); alfredo.vannacci@unifi.it (A.V.); 2 Tuscan Regional Center of Pharmacovigilance, 50122 Florence, Italy; 3 Department of Experimental and Clinical Medicine, University of Florence, 50134 Florence, Italy; alessandra.bettiol@unifi.it; 4 Internal Medicine, Medical Department, Vimercate Hospital, ASST di Vimercate, 20871 Vimercate, Italy; silvia.pagani@asst-brianza.it (S.P.); giulia.spada@asst-brianza.it (G.S.); giudittavioletta.vighi@asst-brianza.it (G.V.V.); giuseppedanilo.vighi@asst-vimercate.it (G.D.V.); 5 Joint Laboratory of Technological Solutions for Clinical Pharmacology, Pharmacovigilance and Bioinformatics, University of Florence, 50139 Florence, Italy; 6 Pharmacology Unit, Department of Diagnostics and Public Health, University of Verona, 37100 Verona, Italy; mauro.venegoni@gmail.com

**Keywords:** pharmacovigilance, clinical pharmacology, male, female, emergency department

## Abstract

This post hoc analysis of an Italian active pharmacovigilance study describes pharmacological differences of ADEs leading to emergency department (ED) visits and hospitalization in women and men. During the study period (January 2007–December 2018), 61,855 reports of ADEs leading to ED visits were collected. Overall, 30.6% of ADEs resulted in hospitalization (30% in women and 31% in men). Multivariate logistic regression showed that, among women, drug classes significantly associated with an increased risk of hospitalization were heparins (ROR 1.41, CI 1.13–176), antidepressants (ROR 1.12, CI 1.03–1.23) and antidiabetics (ROR 1.13, CI 1.02–1.24). Among men, only vitamin K antagonists (ROR 1.28, CI 1.09–1.50), opioids (ROR 1.30, CI 1.06–1.60) and digitalis glycosides (ROR 1.32, CI 1.09–1.59) were associated with a higher risk of hospitalization. Overall, older age, multiple suspected drugs and the presence of comorbidities were significantly associated with a higher risk of hospitalization. A significantly reduced risk of hospitalization was observed in both women and men experiencing an adverse event following immunization (ROR 0.36, CI 0.27–0.48 and 0.83, 0.42–0.74, respectively) compared to drugs. Results obtained from this real-world analysis highlight important aspects of drug safety between sexes.

## 1. Introduction

Women and men are characterized by significant differences in terms of adverse drug event (ADE) occurrence derived from a heterogeneous set of factors in which both intrinsic and extrinsic elements concur [1,2]. Among these: (a) differences between men and women in body–water, muscle/fat mass ratio, organ blood flow and function; (b) physiological aspects such as menopause, pregnancy and menstruation; (c) prevalence of diseases and, consequently, in prescription patterns; (d) the possible impact of genetics and hormonal variations in response to drugs [3,4,5,6]. All these factors describe the complexity of gender pharmacology.

As for randomized clinical trials, exclusion of women and limitation in drug safety evaluation are well-known issues [7,8]. Moreover, characterization of ADEs leading to emergency department (ED) admission in men and women is still lacking. Estimates of ADE risk between the two sexes from observational real-world data, especially from active pharmacovigilance studies, may represent the best strategy to fill this gap [9].

The MEREAFaPS Study was the first national active pharmacovigilance study performed in Italy. The study was based on electronic ED medical records with detailed information on patient populations, which allowed for consideration of risk predictors and modifying factors of ADEs and ADE-related hospitalization, such as polypharmacy and comorbidity, as well as sociodemographic characteristics [9]. This post hoc analysis of data retrieved from the MEREAFaPS Study database aimed to analyze pharmacological characteristics of ADEs leading to ED visits in women and men and to estimate differences in risk of hospitalization by different suspected drug classes.

## 2. Results

### 2.1. Case Characteristics

Between 1 January 2007 and 31 December 2018, 61,855 reports of ADEs leading to ED visit were collected: 35,010 (56.6%) ADE reports for women and 26,845 (43.4%) for men.

Table 1 shows the characteristics of cases. Overall, the majority of patients were Caucasian adults aged 20–79 years, with a median age of 62.4 years for women and 63.8 years for men. In both men and women, the majority of reports were related to drugs, and ≥5 suspected drugs (polypharmacy) were reported in 23.2% of ADE reports for women and 25.4% of ADE reports for men.

A statistically significant difference between the two sexes was observed for all the most frequently reported suspected ATC drug classes. For both sexes, the most frequently reported ATC classes of suspected drugs were antithrombotic agents (16.6% in women and 15.5% in men), followed by antibacterials (16.3% vs. 15.2%), anti-inflammatory and antirheumatic products (8.6% vs. 8.2%), psycholeptics (8.4% vs. 6.0%), analgesics (8.1% vs. 5.6%) and antidiabetics (6.9% vs. 8.7%).

The presence of concomitant drugs was reported in 48% of cases for women and 49% of cases for men. For both sexes, the most frequently reported ATC classes of concomitant drugs were renin–angiotensin system inhibitors (9.7% vs. 10.2%), followed by drugs for acid related disorders (8.3% vs. 8.0%), diuretics (8.0% vs. 7.8%), antithrombotic agents (7.9% vs. 8.4%) and beta blocking agents (7.4% vs. 8.0%).

Comorbidities were reported in 36.4% of cases for women and 37.9% of cases for men, with 10.9% of women and 12.2% of men affected by three or more concomitant diseases. For both sexes, the most reported comorbidity was hypertension, followed by diabetes and atrial fibrillation.

A statistically significant difference between the two sexes was observed for ADEs caused by abuse/misuse, drug–drug or herb–drug interactions, overdose and therapeutic errors.

Overall, 18,918 (30.5%) ADEs caused patient hospitalization (30% in women and 31% in men, *p*-value = 0.042).

### 2.2. Hospitalization among Both Sexes

A statistically significant increased risk of hospitalization was observed for both women and men exposed to antihemorrhagics, antianaemic and perfusion preparations (ROR 1.32, 95% CI 1.14–1.52 and 1.33, 1.13–1.56, respectively); sedative or hypnotic agents (ROR 1.11, 1.01–1.21 and 1.15, 1.02–1.31), particularly benzodiazepines; antipsychotics (ROR 1.57, 1.38–1.79 and 1.44, 1.22–1.68); antiepileptics (ROR 1.23, 1.10–1.39 and 1.21, 1.05–1.38); diuretics (ROR 1.25, 1.15–1.36 and 1.16, 1.06–1.26) and, for diabetes agents, insulin (ROR 1.32, 1.12–1.55 and 1.19, 1.01–1.41) (Table 2).

A significantly reduced risk of hospitalization was observed for both women and men exposed to renin–angiotensin system inhibitors (ROR 0.89, 0.83–0.95 and 0.91, 0.84–0.98, respectively), particularly angiotensin II receptor blockers (plain and combinations) and ibuprofen (ROR 0.52, 0.36–0.76 and 0.56, 0.34–0.90) (Table 2).

### 2.3. Hospitalization Risks among Women

A significantly increased risk of hospitalization was observed only for women when exposed to unfractionated and low-molecular-weight heparins (ROR 1.41, 1.13–1.76), antidepressants (ROR 1.12, 1.03–1.23) and diabetes agents (ROR 1.13, 1.02–1.24) (Table 2).

A statistically significant reduced risk of hospitalization was observed only for women when exposed to analgesics (ROR 0.85, 0.75–0.96), particularly nonopioid analgesics; antibacterials (ROR 0.79, 0.67–0.94), particularly penicillins; beta blocking agents (ROR 0.92, 0.85–0.99); nonsteroidal anti-inflammatory drugs (ROR 0.66, 0.55–0.79), particularly ketorolac; and corticosteroids for systemic use (ROR 0.87, 0.77–0.99) (Table 2).

### 2.4. Hospitalization Risks among Men

A statistically significant increased risk of hospitalization was observed only for men when exposed to vitamin K antagonists (ROR 1.28, 1.09–1.50), opioid analgesics (ROR 1.30, 1.06–1.60), and digitalis glycosides (ROR 1.32, 1.09–1.59) (Table 2).

A significantly reduced risk of hospitalization was observed only for men when exposed to antiplatelets (ROR 0.81, 0.74–0.88), particularly acetylsalicylic acid and platelet P2Y_12_ receptor antagonists (Table 2).

### 2.5. Predictors of Hospitalization

A statistically significant increased risk of hospitalization was observed for both elderly women and men (ROR 1.35, 95% CI 1.27–1.42 and 1.23, 1.16–1.31, respectively) compared to adults, in patients treated with more than one suspected drug (two suspected drugs ROR 1.98, 1.81–2.17 and 1.66, 1.50–1.84; three to four suspected drugs ROR 4.14, 3.66–4.69 and 2.92, 2.53–3.38; five or more drugs ROR 6.58, 5.66–7.66 and 4.15, 3.48–4.96), and in patients who presented concomitant conditions (ROR 1.51, 1.44–1.59 and 1.57, 1.48–1.66) (Table 3).

A significantly increased risk of hospitalization was observed only for men of Black or African American ethnicity (ROR 1.43, 1.08–1.88) compared to Caucasians (Table 3).

A significantly reduced risk of hospitalization was observed for both male and female children and adolescents (ROR 0.85, 0.77–0.95 and 0.56, 0.50–0.64, respectively) compared to adults, and for patients who experienced an adverse event following immunization (ROR 0.36, 0.27–0.48 and 0.83, 0.42–0.74) (Table 3).

### 2.6. Adverse Events

Table 4 shows the description of ADEs according to the most commonly reported suspected drug classes (see Table 1). A statistically significant difference between women and men was observed for the following ADEs: (1) haemorrhages, alteration of the international normalized ratio and unintentional or intentional overdose in patients exposed to anticoagulants and antiplatelets; (2) dermatologic reactions, gastrointestinal disturbances, neurological effects and anaphylaxis due to antibacterials; (3) dermatologic reactions, gastrointestinal disturbances, localized or peripheral edema and abuse or self-harm due to nonsteroidal anti-inflammatory drugs; (4) hypoglycaemia and gastrointestinal disturbances due to insulin and oral antidiabetic agents; (5) neurological effects, gastrointestinal disturbances, dermatologic reactions, localized or peripheral edema and unspecified hypersensitivity due to opioid and nonopioid analgesics. Details of each ADE manifestation are also reported (*italics*).

### 2.7. Suspected Drugs

Supplementary Table S1 shows the most commonly reported suspected drugs by patients’ age. Overall, the most commonly reported drug was warfarin, followed by amoxicillin/clavulanate, acetylsalicylic acid, ketoprofen and ibuprofen. In patients aged ≤19 years old, the most commonly reported drug was amoxicillin/clavulanate, followed by ibuprofen; amoxicillin, alone; hexavalent vaccine and paracetamol. Among elderly patients (age ≥ 65 years), the most commonly reported drug was warfarin, followed by acetylsalicylic acid, amoxicillin/clavulanate, long-acting insulin glargine and furosemide.

## 3. Discussion

The primary aim of our study was to give an overview of differences in ADE-related hospitalization by the most frequently reported suspected drug classes in women and men in Italy. This post hoc analysis showed a higher frequency of ED admission in women, although for this group the frequency of hospitalization resulted lower than in men. This evidence are comparable to those already available in literature, both at the Italian [10,11] and international level [12,13].

Our data reported that men were exposed to polypharmacy (≥5 suspected drugs along with ≥5 concomitant drugs) more frequently than women at the time of ED admission, also presenting more than three comorbidities compared to women. This could be a possible explanation of the higher rate of hospitalization observed among men in our sample. In fact, at a global level, the proportion of serious and fatal reports also associated with hospitalization is higher for males [14].

It is noteworthy that vaccination safety was confirmed by the evidence that the majority of ADE reports were not associated to vaccines, nor was increased risk of hospitalization due to AEFI occurrence was observed among sexes. Although vaccines represent one of the most frequent cause of ADE in specific subgroups, such as children, their safety was confirmed by several observational studies and by health care authorities: adverse events following immunization are mostly nonserious and rarely cause ED visits and hospitalization [15].

According to the study by McHugh and colleagues [16], abuse/misuse and overdoses were more frequently reported among women. A possible explanation of these real-world evidences may be related to the higher prevalence of use of analgesics (i.e., opioids) and sedatives and hypnotics (i.e., benzodiazepines) among women in Italy [17], which could also be confirmed by the higher number of ADE reports related to these suspected drug classes for women in our sample. Furthermore, it is well-known that opioids and benzodiazepines have a high tolerance and dependence potential [18].

On the other hand, interactions (particularly drug–drug and drug–disease interactions) and therapeutic errors were more frequently reported among men. It is well-known that men, particularly the elderly or those exposed to polypharmacy as in our sample, take less care of themselves and, therefore, could be exposed to a greater risk of therapeutic errors, especially in absence of a caregiver, whose lacking is known to be more common among men [19]. Moreover, women more often act as caregivers, and the effect of having a caregiver appears less important for women compared to men [20].

Considering predictors of hospitalization, except for vaccines discussed above, our study confirms a higher risk of hospitalization for both sexes in old age (≥65 years) and in subjects exposed to more than one suspected drug [21]. Moreover, even if genetic differences in drug response related to ethnicity are well established [22,23], social and economic status may have influenced the likelihood of seeing African American ethnicity associated with an increased risk of hospitalization for men. In fact, as reported by an Italian investigation, low education level, lack of employment and negative self-perceived economic resources were conditions associated with the risk of hospitalization, a longer hospital stay and greater recourse to urgent hospitalization [24].

As a final consideration, the majority of ADE manifestations observed in both sexes were represented by dose-dependent and preventable events (Type A reactions), which are usually associated with pharmacodynamic and pharmacokinetic properties of each suspected drug class, and by hypersensitivity events (Type B reactions), whose frequency is higher for antibacterials for systemic use [25].

### 3.1. Drug Classes and Hospitalization Risk among Both Women and Men

#### 3.1.1. Increased Hospitalization

Scientific literature provides several studies concerning antipsychotic safety in women and men [26]. In much research, some side effects, such as weight gain, passivity, hypotension and hyperprolactinemia, are reported to be particularly problematic for women [27]. Nevertheless, in our sample, both women and men are at risk of hospitalization if exposed to antipsychotics. In fact, men and women may experience different ADEs: metabolic abnormalities, hypertension and cardiovascular risk are more frequently reported in men, while women are at a higher risk for gaining weight, developing diabetes and needing laxatives [28].

According to Landmark and Johannessen, there are no suggestion of sex-related influences on antiepileptics pharmacokinetics. In particular, sex does not influence antiepileptics efficacy and safety to a clinically relevant degree. Nevertheless, the use of enzyme inducers or inhibitors, such as carbamazepine or valproate, may be considered with caution, regardless of the patient’s sex [29].

In contrast with our results, the German Pharmacovigilance Project found higher rates of hospitalization due to electrolyte disturbances and arrhythmias in women than in men treated with diuretics. According to the authors, even if dose adjustments are not required for patient’s sex, diuretics elimination in women is reduced, leading to a higher frequency of hyponatraemia and hypokalaemia [30]. Results highlighted by our analysis may derive from a country-specific prescription patterns of diuretics, particularly high-ceiling ones. In fact, in the last report of the Italian Medicine Agency, no relevant differences were observed in diuretics prevalence of use in the general population [17]. Moreover, the clinical impact of concomitant diseases on hospitalization risk should also be considered.

To date, few studies focused on sex differences in the treatment of diabetes mellitus (Type 2) [31]. In line with our results, examination of the antidiabetic ADE pairs by drug class showed high rates of GLP-1RA-, insulin-, and SGLT2i-related reporting in both women and men. Explanations for possible different ADEs reporting by gender in diabetic subjects should be further explored [32,33].

#### 3.1.2. Decreased Hospitalization

Results regarding the risk of hospitalization related to the use of renin–angiotensin system inhibitors and angiotensin II receptor blockers obtained in our sample are strengthened by the evidence of no sex-related differences in the pharmacokinetics of these drugs. Even if the premenopausal cardio-protective effects of estrogen may result in part from renin–angiotensin–aldosterone system inhibition, the efficacy and safety of medications acting on this system seems not to be imbalanced between the two sexes [30].

Compared with other nonsteroidal anti-inflammatory agents, ibuprofen shows a favourable safety profile. The majority of ADEs may be described as gastrointestinal and cardiovascular, but their incidence is relatively rare in both women and men [34]. Moreover, the menstrual cycle did not affect the pharmacokinetics of S-ibuprofen or R-ibuprofen, and only the concomitant administration of oral contraceptives may lead to a higher clearance. Nevertheless, the clinical impact of such interaction seems of little relevance [35].

### 3.2. Drug Classes and Hospitalization Risk among Women

#### 3.2.1. Increased Hospitalization

Our results regarding unfractionated and low-molecular-weight heparins are confirmed by Blanco-Molina et al., who showed an increased rate of major bleeding events in women, irrespective of the active principle used. The authors attributed the higher rate of bleeding in women to their older age, lower body weight or to the higher dosage, which may contribute to reach higher plasma concentration of heparins and increase the risk of ADEs [30,36].

Despite depression’s prevalence in women, the vast majority of research focused on depression has been dedicated to studying males. Different gastric environment, slower gastric emptying and longer colonic transit observed in women can increase antidepressants absorption. In addition, a higher percentage of adipose tissue in women can prolong the half-life of lipophilic drugs. Differences in metabolism or clearance may contribute to higher plasma concentrations in women, and estrogen is a substrate for some of the same cytochrome P450 isozymes as well as antidepressants, possibly shifting their metabolism [30]. Thus, women show a decreased tolerability of such antidepressants, which could lead to dizziness, nausea, abnormal vision, constipation and somnolence [37].

While insulins increased hospitalization risk both in women and men, in our sample, oral antidiabetics related-ADEs were more frequently reported only in women. This is in line with previous evidences that some antidiabetics (i.e., thiazolidinediones) double the risk of fractures among diabetic women but not among men [30]. However, in the study by Rodenburg et al., hypoglycaemic coma due to insulin and antidiabetic agents were more frequent in men [38], whereas Hendriksen and colleagues found that hypoglycaemia was significantly lower in women than in men [13].

#### 3.2.2. Decreased Hospitalization

Concerning nonopioid analgesics, mainly represented by paracetamol in our sample, we observed a reduced risk of hospitalization in women compared to men. This is in contrast with the review by Tamargo et al., which reported that paracetamol overdose and consequent acute liver failure are more common in women. In fact, men show lower plasma levels and higher clearance due to increased activity of the glucuronidation pathway [30].

Several studies from United States and Europe showed that antibiotics prescription is higher in women, although well-known risk factors for bacterial infections (i.e., alcohol drinking, smoking and obesity) are more prevalent in men [39,40]. Despite this, our analysis showed a lower risk of hospitalization due to antibiotic-related ADEs in this group. It can be assumed that women prescribed with antibiotics are more likely to receive appropriate prescriptions and to follow their therapy correctly [41].

Estrogen and progesterone inhibit the cardiac expression of β1-adrenoceptors and reduce β-adrenergic-mediated stimulation. This cardio-protective effect may lead to sex-specific differences in beta blockers pharmacodynamics [40]. This evidence may explain our results concerning beta blockers safety profile in women. In fact, in women, more ADEs were observed for CYP 2D6-dependent beta blockers (i.e., metoprolol, carvedilol, nebivolol and propranolol), suggesting a possible increasing in hospitalization risk in case of drug–drug or drug–herb interactions [42].

Women receive diuretics more often than men, suggesting possible sex-related differences in the treatment of hypertension [40]. In contrast with our analysis, Rodenburg et al. found that women were more frequently admitted to the hospital with an ADE related to high-ceiling and low-ceiling diuretics and cardiotonic glycosides than men [43]. However, no information was reported in this study respect to hospitalization. Therefore, we cannot exclude that, although women are more exposed to diuretics and are admitted more frequently to ED, ADE seriousness in women may not require hospitalization.

### 3.3. Drug Classes and Hospitalization Risk among Men

#### 3.3.1. Increased Hospitalization

As a result of their mechanism of action, anticoagulants cause different types of haemorrhages. Rodenburg et al. showed that the risk of a hospital admission differs between women and men for different types of bleeding. As in our study, men were more at risk for ED visits, in particular caused by unspecified and recurrent and persistent haematuria, haemoptysis and subdural haemorrhage [13]. Warfarin efficacy in reducing the risk of thromboembolism in women and men did not differ and did not pose women at a greater risk of major haemorrhagic complications. Women had more minor bleeding complications than men did, and they require less mg per week than men to maintain a therapeutic International Normalized Ratio, with older women requiring the lowest doses [40]. Another study confirms that the risk of hospitalizations for bleeding in any specific form due to anticoagulants or salicylates use was significantly higher in men. In particular, hospitalizations for haematuria and haemoptysis were much more frequent in men than in women. Risk for ADE-related hospitalization varied per type of reaction. Where men seemed to have a higher risk of hospitalization for haematuria, haemoptysis, cerebral bleeding and bone fractures [44], women were at higher risk for anaemia [38]. Of note, these studies considered anticoagulants and antiplatelets as a unique drug class, even if their pharmacological properties are not comparable.

Regarding opioids, women experience more ADEs (i.e., nausea and vomiting, respiratory depression) despite smaller dose requirements for pain control [38,40]. In our sample, a higher risk of hospitalization was not observed in women but was in men, according to the study by Hendriksen et al., which showed a small difference in the number of hospital admission in favour of men [13].

From our analysis, risk of hospitalization due to cardiotonic glycosides was significantly increased in men. According to the evidences published in literature, women have higher serum digoxin concentrations due to reduced distribution volume and lower clearance that increases only during pregnancy [40]. When directly compared, cardiotonic glycosides are responsible for a twofold higher risk for ED visits in women than men [43] and accounted for a risk for women to be hospitalized with an ADE that was twice as high as for men [43]. Our study did not underline an increased risk for hospitalization in women but, in line with the above cited articles, highlighted a worse safety profile for cardiotonic glycosides in men.

#### 3.3.2. Decreased Hospitalization

As for antiplatelets, the majority of studies available in literature on this topic calculated pooled risk for anticoagulants and antiplatelets as combined drug classes. Following this approach, ED visits for bleeding due to anticoagulants or salicylates use were significantly higher in men [38]. However, when antiplatelets, alone, were considered, more frequent and severe bleedings were observed in women [40]. Again, our study did not underline an increased risk for hospitalization in women but confirmed a better safety profile for antiplatelets in men.

### 3.4. Limitations and Strengths

Main limitations and strengths of the MEREAFaPS Study have already been described [9,45]. For this specific post hoc analysis, limitations are mainly represented by the lack of information on drug use and regarding drug–drug combinations and interactions. Points of strength include minimization of reporting bias by investigating only ADE-related ED visits and hospitalization and the availability of individual patient information, which enabled us to adjust for several demographic and clinical variables. Finally, to the best of our knowledge, this is the first analysis of its kind investigating drug safety in women and men in the setting of the ED without a direct comparison between the two groups. This methodological approach avoids obtaining estimates biased by all the well-known biological differences between sexes.

## 4. Materials and Methods

This is a post hoc analysis performed on pharmacovigilance reports of suspected AE collected between 1 January 2007 and 31 December 2018 in the EDs participating to the MEREAFaPS Study [9,45,46,47].

As described in previous publications [48,49], all ADEs leading to ED visits were collected from the ED clinical charts and hospitalization data were collected from the hospitals discharge database. Patients who developed an ADE while in the ED were excluded. Trained monitors recorded: (1) patients’ demographic characteristics; (2) patients’ clinical status on ED visits; (3) suspected and concomitant drugs; (4) ADEs description.

Suspected and concomitant drugs were classified according to the Anatomical Therapeutic Chemical (ATC) classification system. ADE description according to diagnosis and symptoms was coded using the Medical Dictionary for Regulatory Activities (MedDRA) and organized by System Organ Class (SOC) and Preferred Term (PT) [48]. As already described in previous publications, “suspected” drugs were defined as those mainly associated with the reported ADE, while “concomitant” drugs as those described in the report form at the time of ADE manifestation, which were used by the patient concomitantly with the suspected drugs, but which were not considered directly associated with the ADE. Number of concomitant drugs “0” means that patient was not taking concomitant drugs, but only 1 or more suspected drugs; “1” means that patient was taking only 1 concomitant drug with at least 1 suspected drug; “2” or “≥3” means that patient was taking 2 or more than 3 concomitant drugs with at least 1 suspected drug.

Descriptive statistics were used to summarize data. Categorical data were reported as frequencies and percentages and compared using the chi-square test, whereas continuous data were reported as median values with the related interquartile ranges (IQR) and compared using the Mann–Whitney test. Univariate logistic regression was used to estimate the reporting odds ratios (RORs) of hospitalization with 95% confidence intervals (CIs). In order to reduce bias due to biological differences between women and men, comparisons were made within each sex group rather than between the two sexes. For example, estimating the hospitalization risk associated to anticoagulants, women exposed to these suspected drugs were compared to not exposed ones. The same contingency was built for men. Multivariate logistic regression was performed and adjusted for: age, ethnicity, number of suspected drugs, presence of concomitant drugs and presence of concomitant conditions. All results were considered to be statistically significant at *p*-value < 0.05. Data management and statistical analysis were carried out using STATA 16.1.

The coordinating centre of Tuscany Region (Italy) approved MEREAFaPS Study (Notification number 1225—21 December 2009), and the local institutional ethics committee approved the MEREAFaPS Study (Study number 3055/2010, Protocol number 45288—6 August 2014) according to the legal requirements concerning observational studies. Due to the retrospective nature of the present study and data anonymization, patient consent to participate was not required.

## 5. Conclusions

Results obtained from this real-world analysis highlight important aspects of drug safety between sexes. Healthcare professionals, particularly physicians operating in ED and clinical pharmacologists, should always consider differences in drug safety among women and men, considering a personalized approach for each group in terms of prescription appropriateness and ADE management and prevention.

## Figures and Tables

**Table 1:** Case characteristics.

	ED Visits for ADEs		
	Women	Men	*p*-Value
	*N* = 35,010 (%)	*N* = 26,845 (%)	
**Patient Age, Years**			
≤5	1510 (4.3)	1701 (6.3)	<0.001
6–19	1607 (4.6)	1194 (4.5)	
20–64	15,238 (43.5)	10,801 (40.2)	
65–79	8456 (24.2)	7610 (28.4)	
≥80	7880 (22.5)	5295 (19.7)	
Not Available	319 (0.9)	244 (0.9)	
Median (IQR), Years	62.4 (39.6–78.9)	63.8 (40.8–77.9)	0.108
**Patients’ Ethnicity**			
Asian	467 (1.3)	425 (1.6)	0.005
Black or African American	291 (0.8)	259 (1.0)	
Caucasian	30,729 (87.8)	23,503 (87.6)	
Other	102 (0.3)	54 (0.2)	
Not available	3421 (9.8)	2604 (9.7)	
**Type of Drug**			
Drug	34,425 (98.3)	26,259 (97.8)	<0.001
Vaccine	585 (1.7)	586 (2.2)	
**No. of Suspected Drugs Involved in the ADE**			
1	14,948 (42.7)	11,054 (41.2)	<0.001
2	5937 (17.0)	4575 (17.0)	
3–4	5988 (17.1)	4409 (16.4)	
≥5	8137 (23.2)	6807 (25.4)	
**Most Frequently Reported Suspected ATC Drug Classes**	***N* = 44,119**	***N* = 34,242**	
Antithrombotic Agents (B01)	7322 (16.6)	8732 (15.5)	<0.001
Antibacterials (J01)	7203 (16.3)	5186 (15.2)	<0.001
Anti-inflammatory and Antirheumatic Products (M01)	3805 (8.6)	2812 (8.2)	0.039
Psycholeptics (N05)	3717 (8.4)	2052 (6.0)	<0.001
Analgesics (N02)	3559 (8.1)	1915 (5.6)	<0.001
Diabetes agents (A10)	3051 (6.9)	2991 (8.7)	<0.001
**Presence of a Suspected Drug with Parenteral Administration**			
No	30,478 (87.1)	23,187 (86.4)	0.013
Yes	4532 (12.9)	3658 (13.6)	
**Concomitant Drugs**			
No	18,221 (52.1)	13,691 (51.0)	0.010
Yes	16,789 (48.0)	13,154 (49.0)	
No. of Concomitant Drugs *			
0	18,221 (52.1)	13,691 (51.0)	<0.001
1	4100 (11.7)	2977 (11.1)	
2	2980 (8.5)	2220 (8.3)	
3–4	4414 (12.6)	3343 (12.5)	
≥5	5295 (15.1)	4614 (17.2)	
**Most Frequently Reported Concomitant ATC Drug Classes**	***N* = 61,184**	***N* = 50,843**	
Renin–Angiotensin System Inhibitors (C09)	5943 (9.7)	5175 (10.2)	0.009
Drugs for Acid Related Disorders (A02)	5063 (8.3)	4090 (8.0)	0.162
Diuretics (C03)	4899 (8.0)	3987 (7.8)	0.311
Antithrombotic Agents (B01)	4851 (7.9)	4273 (8.4)	0.004
Beta Blocking Agents (C07)	4521 (7.4)	4040 (8.0)	<0.001
**Presence of Comorbidities**			
No	22,261 (63.6)	16,684 (62.2)	<0.001
Yes	12,749 (36.4)	10,161 (37.9)	
**No. of Comorbidities**			
0	22,261 (63.6)	16,684 (62.2)	<0.001
1	6111 (17.5)	4641 (17.3)	
2	2819 (8.1)	2243 (8.4)	
≥3	3819 (10.9)	3277 (12.2)	
**Most Frequently Reported Comorbidities**			
Hypertension	3892 (14.2)	3261 (14.3)	<0.001
Diabetes	1391 (5.0)	1306 (5.7)	
Atrial Fibrillation	1321 (4.8)	1207 (5.3)	
Allergic Disease	1060 (3.8)	577 (2.4)	
Heart Disease	1023 (3.7)	1413 (6.1)	
Depression	1020 (3.7)	372 (1.6)	
Dyslipidaemia	828 (3.0)	776 (3.4)	
Renal Failure	808 (2.9)	943 (4.09)	
**Presence of CAM**			
No	34,558 (98.7)	26,569 (99.0)	0.003
Yes	452 (1.3)	276 (1.0)	
**Type of Event**			
Abuse/Misuse	1024 (2.9)	550 (2.1)	<0.001
Interactions	281 (0.8)	258 (1.0)	0.036
Overdose	240 (0.7)	147 (0.6)	0.031
Therapeutic Errors	433 (1.2)	399 (1.5)	0.008
**Hospitalization**			
No	24,418 (69.8)	18,519 (69.0)	0.042
Yes	10,592 (30.3)	8326 (31.0)	

ADE: adverse drug event; ATC: anatomical therapeutic chemical; CAM: complementary and alternative medicines; ED: emergency department; IQR: interquartile range. * Number of concomitant drugs: “0” means that patient was not taking concomitant drugs, but only 1 or more suspected drugs; “1” means that patient was taking only 1 concomitant drug with at least 1 suspected drug; “2” or “≥3” means that patient was taking 2 or more than 3 concomitant drugs with at least 1 suspected drug.

**Table 2:** Suspected drug classes and risk of hospitalization.

	ED Visits for ADEs		ED Visits for ADEs		Adjusted ROR	
			Resulting in Hospitalization		(95% CI)	
	Women	Men	Women	Men	Women	Men
	*N* = 35,010 (%)	*N* = 26,845 (%)	*N* = 10,592 (row %)	*N* = 8326 (row %)		
**Blood and Blood Forming Organs B**						
Anticoagulants (B01AA, B01AB, B01AE, B01AF, B01AX)	1363 (3.9)	1044 (3.9)	672 (57.0)	507 (43.0)	1.13 (1.00–1.26)	1.14 (1.00–1.30)
Vitamin K Antagonists (B01AA)	824 (2.4)	652 (2.4)	414 (55.1)	337 (44.9)	1.12 (0.97–1.29)	1.28 (1.09–1.50)
Factor Xa Inhibitors (B01AF)	77 (0.2)	57 (0.2)	31 (58.5)	22 (41.5)	0.78 (0.49–1.24)	0.79 (0.46–1.36)
Unfractionated and Low-Molecular-Weight Heparins (B01AB)	341 (1.0)	240 (0.9)	181 (61.2)	115 (38.9)	1.41 (1.13–1.76)	1.16 (0.89–1.50)
Direct Thrombin Inhibitors (B01AE)	56 (0.2)	46 (0.2)	25 (61.0)	16 (39.0)	0.96 (0.56–1.64)	0.60 (0.32–1.12)
Antiplatelets (B01AC)	3294 (9.4)	2927 (10.9)	1490 (54.9)	1226 (45.1)	0.92 (0.85–1.00)	0.81 (0.74–0.88)
Acetylsalicylic Acid (B01AC06)	2679 (7.7)	2435 (9.1)	1206 (54.8)	995 (45.2)	0.94 (0.86–1.02)	0.79 (0.72–0.87)
Platelet P2Y_12_ Receptor Antagonists (B01AC04, B01AC05, B01AC22, B01AC24, B01AC25)	689 (2.0)	705 (2.6)	317 (51.5)	299 (48.5)	0.91 (0.78–1.07)	0.81 (0.69–0.95)
Antihemorrhagics, Antianaemic and Perfusion Preparations (B02, B03, B05)	828 (2.4)	680 (2.5)	438 (54.3)	369 (45.7)	1.32 (1.14–1.52)	1.33 (1.13–1.56)
**Nervous System N**						
Analgesics (N02)	1346 (3.8)	775 (2.9)	490 (61.3)	309 (38.7)	0.85 (0.75–0.96)	1.07 (0.91–1.24)
Opioid Analgesics (N02A)	737 (2.1)	405 (1.5)	323 (60.7)	209 (39.3)	0.94 (0.81–1.10)	1.30 (1.06–1.60)
Nonopioid Analgesics (N02B)	615 (1.8)	387 (1.4)	174 (60.8)	112 (39.2)	0.74 (0.61–0.89)	0.85 (0.67–1.07)
Antimigraine Preparations (N02C)	43 (0.1)	10 (0.04)	12 (85.7)	2 (14.3)	0.77 (0.39–1.54)	0.43 (0.09–2.08)
Sedative or Hypnotic Agents (N05B, N05C)	2422 (6.9)	1111 (4.1)	1110 (67.9)	525 (32.1)	1.11 (1.01–1.21)	1.15 (1.02–1.31)
Benzodiazepines (N05BA, N05CD)	2251 (6.4)	1008 (3.8)	1034 (68.6)	473 (31.4)	1.12 (1.02–1.22)	1.15 (1.01–1.32)
Nonbenzodiazepine or Nonbarbiturate Sedatives (N05CF)	200 (0.6)	99 (0.4)	92 (63.5)	53 (36.6)	1.04 (0.78–1.38)	1.33 (0.89–1.99)
Antidepressants (N06A)	2385 (6.8)	1116 (4.2)	1101 (68.0)	519 (32.0)	1.12 (1.03–1.23)	1.08 (0.95–1.23)
Selective Serotonin Reuptake Inhibitors (N06AB)	1489 (4.3)	669 (2.5)	705 (69.7)	306 (30.3)	1.19 (1.07–1.33)	1.07 (0.91–1.26)
Nonselective Serotonin Reuptake Inhibitors (N06AA)	141 (0.4)	73 (0.3)	57 (62.6)	34 (37.4)	1.01 (0.71–1.42)	1.27 (0.79–2.04)
Other Antidepressants (N06AF, N06AG, N06AX)	917 (2.6)	444 (1.7)	425 (66.6)	213 (33.4)	1.05 (0.91–1.20)	1.07 (0.88–1.30)
Antipsychotics (N05A)	1051 (3.0)	673 (2.5)	569 (61.7)	353 (38.3)	1.57 (1.38–1.79)	1.44 (1.22–1.68)
Antiepileptics (N03)	1353 (3.9)	960 (3.6)	637 (58.2)	457 (41.8)	1.23 (1.10–1.39)	1.21 (1.05–1.38)
Anti-Parkinson Drugs (N04)	395 (1.1)	340 (1.3)	192 (54.7)	159 (45.3)	1.06 (0.86–1.30)	0.99 (0.80–1.24)
Other Nervous System Agents (N01, N07)	164 (0.5)	131 (0.5)	65 (53.3)	57 (46.7)	0.90 (0.66–1.25)	1.25 (0.87–1.78)
**Anti-Infectives for Systemic Use J**						
Antibacterials (J01)	740 (2.1)	591 (2.2)	227 (52.8)	203 (47.2)	0.79 (0.67–0.94)	0.92 (0.77–1.10)
Penicillins (J01C)	260 (0.7)	221 (0.8)	61 (45.5)	73 (54.5)	0.62 (0.46–0.83)	1.05 (0.78–1.41)
Quinolones (J01M)	190 (0.5)	179 (0.7)	76 (55.1)	62 (44.9)	1.08 (0.79–1.46)	0.76 (0.56–1.05)
Cephalosporins (J01D)	121 (0.4)	89 (0.3)	45 (52.3)	41 (47.7)	1.00 (0.68–1.48)	1.30 (0.84–2.01)
Macrolides (J01F)	122 (0.4)	81 (0.3)	41 (63.1)	24 (36.9)	0.90 (0.61–1.34)	0.83 (0.50–1.36)
Sulfamethoxazole and Trimethoprim (J01E)	40 (0.1)	37 (0.1)	15 (50.0)	15 (50.0)	0.88 (0.45–1.71)	1.03 (0.53–2.00)
Other Antibacterials (J01A, J01B, J01G, J01R, J01X)	60 (0.2)	29 (0.1)	14 (51.9)	13 (48.2)	0.54 (0.29–0.99)	1.40 (0.66–2.96)
Vaccines (J07)	34 (0.1)	45 (0.2)	3 (50.0)	3 (50.0)	0.37 (0.11–1.23)	0.33 (0.10–1.10)
Antivirals and Antiretrovirals (J05)	91 (0.3)	154 (0.6)	29 (32.6)	60 (67.4)	0.77 (0.49–1.22)	0.95 (0.68–1.33)
Other Anti-Infective Agents (J02, J04, J06)	49 (0.1)	44 (0.2)	20 (58.8)	14 (41.2)	0.98 (0.55–1.77)	0.69 (0.36–1.33)
**Cardiovascular System C**						
Renin–Angiotensin System Inhibitors (C09)	5793 (16.6)	4998 (18.6)	2531 (53.7)	2181 (46.3)	0.89 (0.83–0.95)	0.91 (0.84–0.98)
ACE Inhibitors (Plain and Combinations) (C09A, C09B)	3255 (9.3)	3120 (11.6)	1477 (51.4)	1399(48.6)	0.99 (0.92–1.08)	0.99 (0.91–1.08)
Angiotensin II Receptor Blockers (Plain and Combinations) (C09C, C09D)	2620 (7.5)	1990 (7.4)	1092 (56.4)	843 (43.6)	0.84 (0.77–0.91)	0.88 (0.80–0.98)
Diuretics (C03)	4004 (11.4)	3203 (11.9)	2100 (56.3)	1633 (43.7)	1.25 (1.15–1.36)	1.16 (1.06–1.26)
Low-Ceiling Diuretics (C03A, C03B)	156 (0.5)	120 (0.5)	64 (54.2)	54 (45.8)	0.83 (0.60–1.15)	0.87 (0.60–1.26)
High-Ceiling Diuretics (C03C)	3225 (9.2)	2745 (10.2)	1743 (55.2)	1413 (44.8)	1.29 (1.18–1.41)	1.16 (1.05–1.27)
Beta Blocking Agents (C07)	4482 (12.8)	4004 (14.9)	2000 (52.6)	1805 (47.4)	0.92 (0.85–0.99)	0.94 (0.87–1.03)
Calcium Channel Blockers (C08)	2213 (6.3)	1929 (7.2)	1033 (53.9)	884 (46.1)	0.99 (0.90–1.09)	0.97 (0.88–1.08)
Antiarrhythmics (C01B)	935 (2.7)	989 (3.7)	438 (48.0)	475 (52.0)	1.01 (0.89–1.16)	1.09 (0.96–1.25)
Lipid Modifying Agents (C10)	3029 (8.7)	3361 (12.5)	1311 (46.3)	1519 (53.7)	0.84 (0.77–0.91)	0.92 (0.85–1.01)
Digitalis Glycosides (C01AA)	813 (2.3)	463 (1.7)	425 (62.8)	252 (37.2)	1.10 (0.95–1.27)	1.32 (1.09–1.59)
Antiadrenergic Agents C02CA	435 (1.2)	490 (1.8)	208 (49.9)	209 (50.1)	1.03 (0.85–1.26)	0.84 (0.70–1.02)
Other Cardiovascular Agents (C01, C02, C04, C05, Excluding C01AA and C02CA)	1991 (5.7)	2082 (7.8)	1008 (49.4)	1033 (50.6)	1.13 (1.02–1.25)	1.14 (1.03–1.25)
**Alimentary Tract and Metabolism A**						
Diabetes agents (A10)	1991 (5.7)	1864 (6.9)	961 (52.6)	867 (47.4)	1.13 (1.02–1.24)	1.06 (0.96–1.18)
Insulins (A10A)	662 (1.9)	641 (2.4)	345 (52.3)	315 (47.7)	1.32 (1.12–1.55)	1.19 (1.01–1.41)
Oral diabetes agents (A10B)	1475 (4.2)	1360 (5.1)	692 (53.2)	610 (46.9)	1.04 (0.93–1.16)	0.96 (0.86–1.08)
Antiulcer and antacid agents (A02B, A02A)	4899 (14.0)	3975 (14.8)	2319 (55.3)	1872 (44.7)	1.06 (0.98–1.14)	1.04 (0.96–1.13)
Antiemetics and antinauseants (A03F, A04)	329 (0.9)	152 (0.6)	142 (65.1)	76 (34.9)	1.01 (0.81–1.27)	1.24 (0.89–1.72)
Antidiarrheals (A07)	301 (0.9)	263 (1.0)	114 (51.4)	108 (48.7)	0.81 (0.63–1.03)	0.92 (0.72–1.19)
Drugs for constipation (A06)	260 (0.7)	158 (0.6)	141 (63.2)	82 (36.8)	1.27 (0.99–1.63)	1.21 (0.88–1.67)
Stomatological preparations (A01)	11 (0.03)	7 (0.03)	2 (50.0)	2 (50.0)	0.50 (0.10–2.35)	0.77 (0.14–4.19)
Other gastrointestinal agents (A03, A05, A08, A09, A11, A12, A13, A14, A15, A16, excluding A03F)	928 (2.7)	576 (2.2)	472 (61.9)	291 (38.1)	1.19 (1.04–1.37)	1.18 (0.99–1.40)
**Musculoskeletal system M**						
Nonsteroidal Anti-Inflammatory Drugs (M01A)	608 (1.7)	322 (1.2)	170 (63.4)	98 (36.6)	0.66 (0.55–0.79)	0.83 (0.65–1.07)
Ketoprofen (M01AE03, M01AE53)	103 (0.3)	52 (0.2)	26 (65.0)	14 (35.0)	0.69 (0.44–1.09)	0.82 (0.44–1.54)
Ibuprofen (M01AE01, M01AE51)	172 (0.5)	111 (0.4)	37 (62.7)	22 (37.3)	0.52 (0.36–0.76)	0.56 (0.34–0.90)
Diclofenac (M01AB05, M01AB55)	88 (0.3)	50 (0.2)	36 (65.5)	19 (34.6)	1.12 (0.72–1.74)	1.04 (0.58–1.86)
Nimesulide (M01AX17)	56 (0.2)	24 (0.09)	16 (55.2)	13 (44.8)	0.81 (0.45–1.47)	2.19 (0.96–5.02)
Ketorolac (M01AB15)	55 (0.2)	35 (0.1)	13 (54.2)	11 (45.8)	0.47 (0.25–0.89)	0.72 (0.34–1.48)
Naproxen (M01AE02, M01AE52, M01AE56)	21 (0.06)	8 (0.03)	6 (75.0)	2 (25.0)	0.65 (0.25–1.73)	0.61 (0.12–3.12)
Etoricoxib (M01AH05)	57 (0.2)	16 (0.06)	23 (76.7)	7 (23.3)	0.83 (0.48–1.42)	1.11 (0.41–3.04)
Others (M01AA, M01AC, M01AG, M01AX)	83 (0.2)	32 (0.1)	22 (61.1)	14 (38.9)	0.62 (0.37–1.02)	1.49 (0.72–3.08)
Muscle Relaxants (M03)	112 (0.3)	79 (0.3)	33 (55.9)	26 (44.1)	0.69 (0.46–1.05)	0.72 (0.44–1.17)
Antigout Preparations (M04)	1138 (3.3)	1388 (5.2)	616 (47.1)	692 (52.9)	1.20 (1.06–1.37)	1.08 (0.96–1.22)
Topical Products (M02)	26 (0.07)	20 (0.07)	8 (80.0)	2 (20.0)	0.88 (0.37–2.08)	0.22 (0.05–0.98)
Bisphosphonates (M05)	356 (1.0)	59 (0.2)	145 (82.4)	31 (17.6)	0.83 (0.66–1.03)	1.25 (0.74–2.10)
**Antineoplastic and Immunomodulating Agents L**						
Antineoplastic Agents (L01)	260 (0.7)	144 (0.5)	95 (64.2)	53 (35.8)	0.79 (0.61–1.03)	0.77 (0.55–1.10)
Immune Modulators (L03)	22 (0.06)	23 (0.09)	10 (41.7)	14 (58.3)	1.32 (0.56–3.12)	2.09 (0.90–4.87)
Endocrine Therapy (L02)	220 (0.6)	143 (0.5)	93 (57.1)	70 (42.9)	1.05 (0.80–1.39)	0.98 (0.70–1.37)
**Respiratory System R**						
Nasal, Throat, Cough and Cold Preparations (R01, R02, R05)	192 (0.6)	179 (0.7)	50 (49.5)	51 (50.5)	0.65 (0.47–0.91)	0.70 (0.50–0.98)
Bronchodilators (R03)	891 (2.5)	960 (3.6)	395 (46.0)	464 (54.0)	1.05 (0.91–1.21)	1.18 (1.03–1.36)
Antihistamines for Systemic Use (R06)	258 (0.7)	149 (0.6)	83 (63.4)	48 (36.6)	0.75 (0.57–0.98)	0.82 (0.57–1.17)
**Hormonal Preparations H**						
Corticosteroids for Systemic Use (H02)	1143 (3.3)	654 (2.4)	441 (58.8)	309 (41.2)	0.87 (0.77–0.99)	1.28 (1.09–1.51)
Thyroid Therapy (H03)	2285 (6.5)	675 (2.5)	937 (74.8)	315 (25.2)	0.93 (0.85–1.02)	1.02 (0.87–1.20)
Other Hormonal Agents (H01, H04, H05)	33 (0.09)	29 (0.1)	10 (40.0)	15 (60.0)	0.56 (0.26–1.20)	1.26 (0.60–2.63)
**Genitourinary System and Sex Hormones G**						
Systemic and Vaginal Contraceptives (G01)	14 (0.04)	6 (0.02)	6 (75.0)	2 (25.0)	1.29 (0.43–3.89)	0.91 (0.16–5.23)
Drugs Used in Benign Prostatic Hypertrophy (G04)	60 (0.2)	1953 (7.3)	27 (2.9)	910 (97.1)	0.93 (0.55–1.57)	0.98 (0.88–1.08)
Other Gynaecological Agents and Sex Hormones (G02, G03)	161 (0.5)	41 (0.2)	44 (72.1)	17 (27.9)	0.79 (0.55–1.14)	0.75 (0.40–1.42)

Analyses were adjusted for age, ethnicity, presence of two or more suspected drugs, presence of concomitant drugs and presence comorbidities. ADE: adverse drug event; CI: confidence interval; ED: emergency department; ROR: odds ratio.

**Table 3:** Predictors of hospitalization among women and men expressed as reporting odds ratios.

	Women		Men	
	Crude ROR (95% CI)	Adjusted ROR (95% CI)	Crude ROR (95% CI)	Adjusted ROR (95% CI)
**Age Classes**				
Adults (20–64 Years)	1	1	1	1
Children and Adolescents (0–19 Years)	0.62 (0.56–0.69)	0.85 (0.77–0.95)	0.41 (0.37–0.46)	0.56 (0.50–0.64)
Elderly (≥65 Years)	2.00 (1.90–2.10)	1.35 (1.27–1.42)	1.79 (1.69–1.88)	1.23 (1.16–1.31)
**Ethnicity**				
Caucasians	1	1	1	1
Black or African American	0.79 (0.61–1.03)	1.24 (0.95–1.63)	0.96 (0.74–1.25)	1.43 (1.08–1.88)
Asian	0.57 (0.46–0.72)	0.89 (0.70–1.13)	0.61 (0.48–0.76)	0.93 (0.73–1.19)
Other	0.78 (0.50–1.22)	1.09 (0.69–1.73)	0.63 (0.33–1.19)	0.86 (0.44–1.67)
**AEFI**				
No	1	1	1	1
Yes	0.30 (0.20–0.34)	0.36 (0.27–0.48)	0.27 (0.21–0.35)	0.83 (0.42–0.74)
**Number of Suspected Drugs**				
1	1	1	1	1
2	1.56 (1.45–1.67)	1.98 (1.81–2.17)	1.45 (1.33–1.57)	1.66 (1.50–1.84)
3–4	2.52 (2.36–2.69)	4.14 (3.66–4.69)	2.39 (2.21–2.58)	2.92 (2.53–3.38)
≥5	4.24 (4.00–4.51)	6.58 (5.66–7.66)	3.91 (3.65–4.17)	4.15 (3.48–4.96)
**Concomitant Conditions**				
No	1	1	1	1
Yes	1.98 (1.89–2.07)	1.51 (1.44–1.59)	2.09 (1.98–2.20)	1.57 (1.48–1.66)

Analyses were adjusted for age classes, ethnicity, type of drug, number of suspected drugs, number of concomitant drugs and presence of comorbidities. AEFI: adverse events following immunization; CI: confidence interval; ROR: reporting odds ratios.

**Table 4:** Adverse events manifestation by most commonly reported suspected drug classes.

	ED Visits for ADEs Resulting in Hospitalization		
	Women	Men	*p*-Value
	No. of Preferred Terms	No. of Preferred Terms	
	*N* = 47,937 (%)	*N* = 36,231 (%)	
**Anticoagulants and Antiplatelets (B01A)**			
Haemorrhage	4431 (43.65)	5719 (51.45)	<0.001
*Epistaxis*	*1476 (14.52)*	*2083 (18.83)*	
*Gastrointestinal*	*858 (8.46)*	*1009 (9.13)*	
*Genitourinary*	*394 (3.88)*	*805 (7.28)*	
*Central Nervous System*	*554 (5.46)*	*601 (5.44)*	
*Dermatologic*	*54 (0.53)*	*59 (0.54)*	
*Pulmonary*	*78 (0.77)*	*151 (1.37)*	
*Ophthalmic*	*183 (1.8)*	*195 (1.76)*	
Altered International Normalized Ratio	822 (8.10)	661 (5.97)	<0.001
Anaemia	514 (5.06)	574 (5.19)	0.658
Unintentional or Intentional Overdose	167 (1.65)	145 (1.31)	0.045
**Antibacterials (J01)**			
Dermatologic Reactions	6413 (47.74)	4441 (47.73)	<0.001
*Urticaria*	*1893 (14.10)*	*1315 (14.14)*	
*Localized or General Pruritus*	*1661 (12.38)*	*1038 (11.16)*	
*Erythema*	*1197 (8.91)*	*853 (9.17)*	
*Rash*	*249 (1.85)*	*238 (2.55)*	
Localized or Peripheral Edema	1049 (7.82)	746 (8.03)	0.571
Gastrointestinal Disturbances	1532 (11.43)	913 (9.83)	<0.001
*Nausea or Vomiting*	*700 (5.22)*	*380 (4.09)*	
*Abdominal Pain*	*397 (2.96)*	*247 (2.66)*	
*Diarrhoea*	*351 (2.62)*	*237 (2.55)*	
Unspecified Hypersensitivity	636 (4.74)	414 (4.46)	0.311
Neurological Effects	778 (5.67)	468 (5.03)	0.013
Respiratory Reactions	728 (5.43)	486 (5.22)	0.514
*Dyspnoea*	*405 (3.02)*	*279 (3.0)*	
*Throat Tightness*	*127 (0.95)*	*64 (0.69)*	
Anaphylaxis	117 (0.87)	118 (1.27)	0.004
**Nonsteroidal Anti-Inflammatory Drugs (M01A)**			
Dermatologic Reactions	2220 (32.11)	1571 (30.01)	0.012
*Urticaria*	*768 (11.11)*	*579 (11.06)*	
*Localized or General Pruritus*	*572 (8.28)*	*385 (7.35)*	
*Erythema*	*376 (5.44)*	*278 (5.31)*	
*Rash*	*378 (5.47)*	*244 (4.66)*	
Gastrointestinal Disturbances	1256 (18.18)	862 (16.51)	0.014
*Abdominal Pain*	*497 (7.2)*	*316 (6.04)*	
*Nausea or Vomiting*	*351 (5.08)*	*149 (2.85)*	
*Melena*	*83 (1.2)*	*98 (1.87)*	
*Gastritis*	*91 (1.32)*	*65 (1.24)*	
*Hematemesis*	*58 (0.84)*	*65 (1.24)*	
Localized or Peripheral Edema	949 (13.73)	844 (16.14)	<0.001
Unspecified Hypersensitivity	235 (3.4)	198 (3.79)	0.262
Respiratory Reactions	243 (3.51)	216 (4.13)	0.081
Abuse or Self-Harm	100 (1.45)	52 (0.99)	0.026
**Sedative or Hypnotic Agents (N05B, N05C)**			
Neurological Effects	3721 (57.44)	2049 (56.63)	0.292
*Drowsiness*	*1226 (18.93)*	*607 (16.7)*	
*Altered Mental Status or Bradyphrenia*	*504 (7.78)*	*270 (7.43)*	
*Loss of Consciousness*	*179 (2.76)*	*103 (3.04)*	
*Bradykinesia*	*196 (3.03)*	*119 (3.28)*	
*Muscular Weakness*	*116 (2.56)*	*101 (2.78)*	
*Presyncope or Syncope*	*127 (1.96)*	*74 (2.04)*	
**Opioid and Nonopioid Analgesics (N02A, N02B)**			
Neurological Effects	2124 (28.29)	866 (23.21)	<0.001
*Muscular Weakness*	*167 (2.22)*	*85 (2.28)*	
*Dizziness*	*411 (5.47)*	*147 (3.94)*	
*Presyncope or Syncope*	*258 (3.44)*	*97 (2.6)*	
*Drowsiness*	*204 (2.71)*	*99 (2.65)*	
*Hyperhidrosis*	*115 (1.53)*	*84 (2.25)*	
*Altered Mental Status*	*246 (3.28)*	*117 (3.14)*	
*Headache*	*107 (1.43)*	*32 (0.86)*	
Gastrointestinal Disturbances	2076 (27.65)	725 (19.43)	<0.001
*Nausea or Vomiting*	*1370 (18.25)*	*409 (10.96)*	
*Abdominal Pain*	*508 (6.77)*	*199 (5.33)*	
*Constipation*	*65 (0.86)*	*34 (0.91)*	
Dermatologic Reactions	1011 (13.47)	686 (18.39)	<0.001
*Urticaria*	*343 (4.57)*	*249 (6.68)*	
*Localized or General Pruritus*	*286 (3.81)*	*193 (5.17)*	
*Erythema*	*214 (2.85)*	*125 (3.35)*	
*Rash*	*168 (2.24)*	*119 (3.19)*	
Localized or Peripheral Edema	380 (5.07)	248 (6.64)	0.001
Abuse or Self-Harm	173 (2.31)	89 (2.38)	0.787
Respiratory Distress	152 (2.03)	97 (2.60)	0.051
Unspecified Hypersensitivity	106 (1.41)	91 (2.44)	<0.001
**Insulin and Oral Antidiabetic Agents (A10)**			
Hypoglycaemia (from Mild to Severe)	1476 (42.73)	1530 (46.8)	0.001
Hypoglycaemia-Related Symptoms	620 (26.41)	575 (17.62)	0.699
*Shock, Loss of Consciousness or Seizures*	*260 (7.52)*	*276 (8.45)*	
*Altered Mental Status*	*139 (4.03)*	*129 (3.96)*	
*Presyncope or Syncope*	*72 (2.09)*	*80 (2.45)*	
*Acidosis*	*89 (2.58)*	*46 (1.41)*	
Neurological Effects	432 (12.51)	413 (12.63)	0.876
*Drowsiness*	*114 (3.3)*	*117 (3.58)*	
*Hyperhidrosis*	*88 (2.55)*	*85 (2.60)*	
*Muscular Weakness*	*61 (1.77)*	*41 (1.25)*	
*Aphasia, Dizziness or Tremor*	*63 (1.82)*	*71 (2.17)*	
Therapeutic Errors	152 (4.4)	164 (5.02)	0.233
Gastrointestinal Disturbances	151 (4.37)	98 (3.01)	0.003

ADE: adverse drug event; ED: emergency department.

## Data Availability

Data that support the findings of this study are available upon reasonable request from the corresponding author, N.L.

## Supplementary Materials

The following are available online at https://www.mdpi.com/article/10.3390/ph14070678/s1, Table S1: Most commonly reported suspected drugs by patient’s age.
