# Supplementary material for: Risk of Hospitalization for Adverse Drug Events in Women and Men: A Post Hoc Analysis of an Active Pharmacovigilance Study in Italian Emergency Departments
Source: Pharmaceuticals (Basel). 2021 Jul 15;14(7):678. doi: 10.3390/ph14070678 (PMC8308702; doi:10.3390/ph14070678)
Supplement: Supplementary file 1 [file pharmaceuticals-14-00678-s001.zip › pharmaceuticals-1277934-supplementary.pdf]

Supplementary Table 1 - Most commonly reported suspected drugs by patients' age.

	ED Visits for ADEs		ED Visits for ADEs Resulting in Hospitalization	
	Women	Men	Women	Men
Suspected Drug	No. of Suspected Drugs N (%)	No. of Suspected Drugs N (%)	No. of Suspected Drugs N (row %)	No. of Suspected Drugs N (row %)
**All patients (N = 18,918 ED visits leading to hospitalized out of 61,855)**	**N=44,119**	**N=34,242**		
Warfarin (B01AA03)	3,640 (8.3)	3,681 (10.8)	1317 (36.2)	1294 (35.2)
Amoxicillin and beta-lactamase inhibitor (J01CR02)	3,013 (6.8)	2,304 (6.7)	540 (17.9)	358 (15.5)
Acetylsalicylic acid (B01AC06)	1,641 (3.7)	2,512 (7.3)	611 (37.2)	912 (36.3)
Ketoprofen (M01AE03)	995 (2.3)	881 (2.6)	216 (21.7)	202 (22.9)
Ibuprofen (M01AE01)	936 (2.1)	671 (2.0)	204 (21.8)	129 (19.2)
Amoxicillin (J01CA04)	908 (2.1)	727 (2.1)	149 (16.4)	116 (16.0)
Paracetamol (N02BE01)	735 (1.7)	528 (1.5)	184 (25.0)	124 (23.5)
Levofloxacin (J01MA12)	652 (1.5)	538 (1.6)	174 (26.7)	147 (27.3)
Diclofenac (M01AB05)	631 (1.4)	-	175 (27.7)	-
Codeine, combination excl. psycholeptics (N02AA59)	600 (1.4)	-	149 (24.8)	-
Insulin glargine (A10AE04)	-	593 (1.7)	-	318 (53.6)
Clopidogrel (B01AC04)	-	586 (1.7)	-	231 (39.4)
**Patients aged ≤19 y (N = 1,071 hospitalized out of 6,260)**	**N=3900**	**N=3656**		
Amoxicillin and beta-lactamase inhibitor (J01CR02)	616 (15.8)	610 (16.7)	43 (7.0)	45 (7.4)
Ibuprofen (M01AE01)	221 (5.7)	207 (5.7)	33 (14.9)	25 (12.1)
Amoxicillin (J01CA04)	186 (4.8)	225 (6.2)	12 (6.5)	11 (4.9)
Diphtheria-haemophilus influenzae B-pertussis-poliomyelitis-tetanus-hepatitis B (J07CA09)	148 (3.8)	169 (4.6)	14 (9.5)	21 (12.4)
Paracetamol (N02BE01)	134 (3.4)	141 (3.9)	34 (25.4)	22 (15.6)
Pneumococcus, purified polysaccharides antigen conjugated (J07AL02)	130 (3.3)	129 (3.5)	16 (12.3)	17 (13.2)
Ketoprofen (M01AE03)	111 (2.9)	93 (2.5)	29 (26.1)	14 (15.1)
Measles, combinations with mumps and rubella, live attenuated (J07BD52)	104 (2.7)	125 (3.4)	7 (6.7)	7 (5.6)
Clarithromycin (J01FA09)	94 (2.4)	95 (2.6)	6 (6.4)	13 (13.7)
Cefixime (J01DD08)	64 (1.6)	-	2 (3.1)	-
Diphtheria-pertussis-poliomyelitis-tetanus (J07CA02)	-	91 (2.5)	-	4 (4.40)
**Patients aged ≥65 y (N = 11,296 hospitalized out of 29,241)**	**N=20,767**	**N=16,587**		
Warfarin (B01AA03)	3294 (15.9)	3157 (19.0)	1209 (36.7)	1124 (35.6)
Acetylsalicylic acid (B01AC06)	1382 (6.7)	1972 (11.9)	539 (39.0)	747 (37.9)
Amoxicillin and beta-lactamase inhibitor (J01CR02)	581 (2.8)	328 (2.0)	171 (29.4)	75 (22.9)
Insulin glargine (A10AE04)	399 (1.9)	336 (2.0)	217 (54.4)	202 (60.1)
Furosemide (C03CA01)	377 (1.8)	246 (1.5)	214 (56.8)	135 (54.9)
Metformin (A10BA02)	360 (1.7)	262 (1.6)	212 (58.9)	165 (63.0)
Levofloxacin (J01MA12)	298 (1.4)	245 (1.5)	104 (34.9)	88 (35.9)
Clopidogrel (B01AC04)	273 (1.7)	466 (2.8)	109 (39.9)	193 (41.4)
Acenocoumarol (B01AA07)	272 (1.3)	345 (2.1)	124 (45.6)	144 (41.7)
Codeine, combination excl. psycholeptics (N02AA59)	267 (1.3)	-	74 (27.7)	-
Ramipril (C09AA05)	-	245 (1.5)	-	113 (46.1)

ED: emergency department
